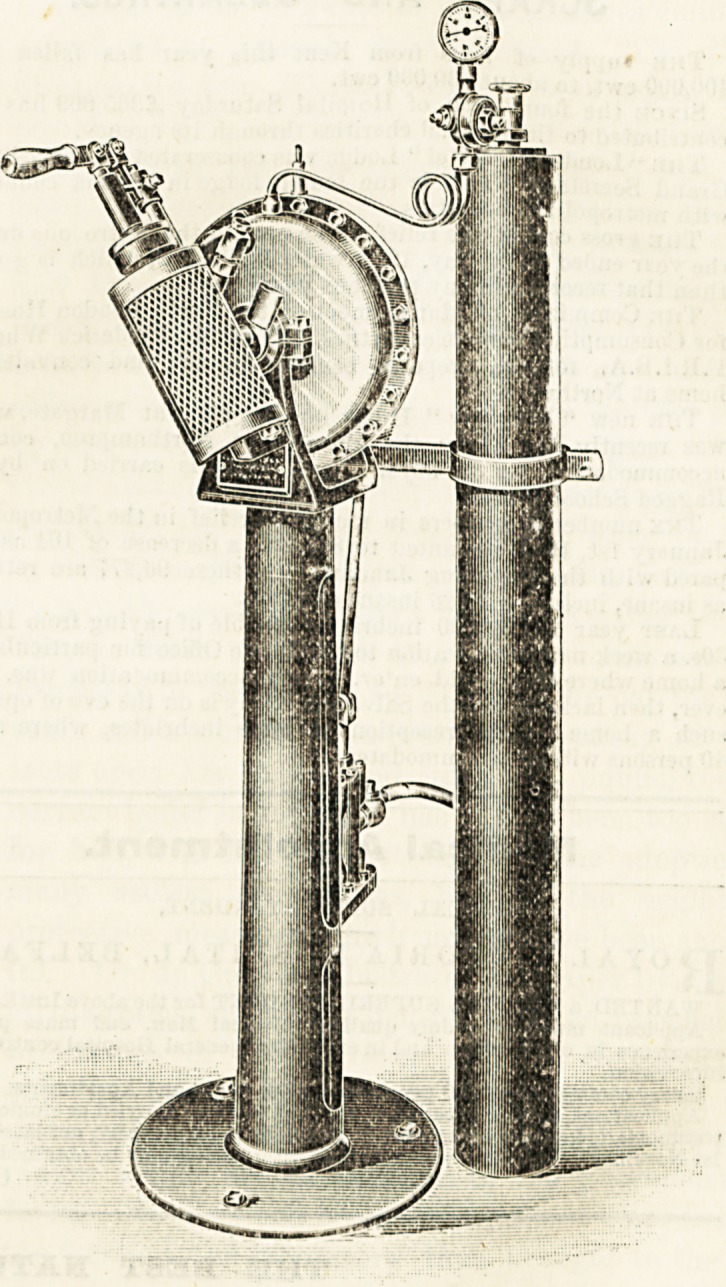# Practical Departments

**Published:** 1901-10-19

**Authors:** 


### PRACTICAL DEPARTMENTS.

### AN INEXPENSIVE MINERAL WATER MACHINE.

A simple and inexpensive machine for making mineral water on the premises should prove a blessing to hospitals and institutions; and one such has just been produced by the "Universal" Mineral Water Machine Company, Limited (Fletcher's Patent). We have seen it at work, and have tasted the lemonade made by it; it was exceedingly good, and very quickly produced. The operator put a few drops of lemon-syrup into a glass bottle, placed the bottle in the "Filler," gave a turn with the handle, and the bottle was ready, filled, and stoppered. In this one turn, we were informed, three separate actions were included, viz.:—

1. The automatic introduction of liquid and gas.
2. Their effective mixture together, termed "aeration."
3. The filling and stoppering of the bottle.

The following is the specification of the machine: It consists of a gun-metal cylinder, 12 inches in diameter and 6 inches wide, fixed on an iron pedestal about 4 feet high, with a circular base in which are screw holes for fastening to the floor. Through the centre of this carbonating cylinder passes a hollow spindle, to which beaters are attached, so as to revolve within the cylinder. At one end of the spindle is affixed a turn-over bottle-filler, to which the aerated liquid is supplied from the cylinder by a tube passing through the spindle. Affixed to the pedestal of the machine is a small pump, the piston of which works on a 7-inch tooth wheel, set in motion by a pinion wheel attached to the other end of the spindle, the pump being connected with the cylinder by a small pipe. With each revolution of the handle (acting through the spindle) fresh liquid is pumped into the cylinder, exactly compensating for the liquid withdrawn and bottled, the stroke of the pump being adjustable to different sized bottles by altering the pin on the wheel, to which the piston-rod is attached. The gas tube is attached to the machine by a bracket clip, and its nozzle connected by a small pipe to the cylinder, a gauge indicating the pressure, which is regulated by an automatic valve. The liquid feeding the pump may be supplied from a filter (either stoneware, or attached to a main) or from any convenient source, provided however that the liquid used is pure and of good quality. Having connected the liquid and turned on the gas, the operator on commencing revolves the handle a few times, until a gauge on the carbonating cylinder indicates that it is pumped half full of liquid. He then inserts a bottle in the bottling arm, and by one revolution it is filled and stoppered; this operation being repeated until all required are filled. From five to eight bottles a minute can easily be made and filled, according to the handiness of the operator. The machine occupies very small space (about 2 feet square) and can be lifted by a man. One of its advantages is that all kinds of aerated and effervescing drinks can be made with it, without necessitating alteration or adjustment. Any liquid may, in fact, be aerated by its means. Another advantage is that a youth can work it single-handed. The price of the machine is £35, and the cost of the aerated waters is estimated at less than one penny per dozen bottles. The gas necessary for aeration is obtainable in tubes, a single one containing sufficient for 250 dozen bottles.

## Figures and Tables

**Figure f1:**